# Dental Extraction Can Be Performed Safely in Patients on Aspirin Therapy: A Timely Reminder

**DOI:** 10.1155/2014/463684

**Published:** 2014-04-01

**Authors:** Gaurav Verma

**Affiliations:** Department of Oral and Maxillofacial Surgery, Himachal Institute of Dental Sciences, Paonta Sahib, Himachal Pradesh 135001, India

## Abstract

Cardiac patients on aspirin therapy may require extractions for their diseased teeth. It is a common practice among physicians and treating surgeons to stop aspirin prior to tooth extraction because of fear of bleeding complications. This practice often predisposes the patient to adverse thromboembolic events. This practice is based on theoretical risk of bleeding and on isolated case reports of excessive bleeding with aspirin therapy. The current consensus and recommendations are in favor of continuing aspirin therapy during simple tooth extraction as the bleeding complication incidence is very less and if it occurs can be controlled efficiently with local hemostasis measures.

## 1. Introduction

Medical practitioners often advise patients on antiplatelet therapy to either stop or alter their medications prior to invasive surgical procedure because of fear of excessive and uncontrolled bleeding. Although there is increased risk of intraoperative and postoperative bleeding if aspirin is continued, there is increased risk of thromboembolic events such as cerebrovascular accidents and myocardial infarction if medication is altered or discontinued [1].

The purpose of this paper is to spread the message that alteration in aspirin schedule should be based on invasiveness of the surgical procedure and must be individualized. A review of dental literature is presented which establishes the safety of dental extractions in patients on aspirin therapy.

## 2. Human Physiology and Functions of Platelets

The blood is a fluid connective tissue, and hemostatic mechanism is chiefly responsible for stopping the extravasation of blood in case of injury to blood vessels. Classically, hemostasis mechanism is characterized by two consecutive phases: primary and secondary. The primary phase primarily involves the vascular and platelet mediated events that help in early arrest of bleeding as a result of platelet plug formation. Secondary hemostasis phase is mediated by complex cascade of clotting factors which help in formation of fibrin clot to ensure definite sealing of ruptured blood vessels. In normal physiological state, perfect balance exists between coexisting interlinked mechanisms like coagulation/anticoagulation and fibrinolysis/antifibrinolysis. Disturbance in the balance in favor of one mechanism results in either bleeding or thrombosis [2].

There is an inbuilt mechanism in human beings which checks against intravascular coagulation and maintained the blood in fluid state intravascularly. These mechanisms are as follows.

1. The rapid flow of blood keeps coagulation factors at lower concentration.
2. Clotting factors intravascularly and thrombin formed in circulation are inactivated by antithrombin III.
3. Fibrinolytic system removes the traces of fibrin formed in the circulation [3].

Platelets are small sized, disk-shaped cells without a nucleus. These are derived from bone marrow and released into the circulation and have an average life span of 10 days. When present in the circulation, platelets have no tendency for adhesion to each other and vessel wall. However, when stimulated, they show alteration in shape, aggregate together, and adhere to the vessel wall. The activation of platelets, aggregation together, and adherence to the vessel wall is an integral part of physiological hemostasis mechanism. Platelets form a platelet plug to occlude the bleeding vessel and help in hemostasis [3].

## 3. Mechanism of Action of Aspirin

The pathological basis related to arterial thrombosis involves atherosclerotic vascular disease mediated by platelet thrombi. Thrombin is a principle mediator in this type of thrombosis. Treatment modality against the arterial thrombosis involves drugs with antiplatelet and antithrombin activity [4].

The antithrombotic effect of aspirin is mediated by irreversible inhibition of cyclooxygenase activity in platelets. On activation, phospholipase-A_2_ acts on the cell membrane to release arachidonic acid. Cyclooxygenase acts on arachidonic acid to produce thromboxane A_2_. Thromboxane A_2_ is a potent platelet stimulant leading to degranulation of platelet and platelet aggregation. Aspirin inhibits cyclooxygenase enzyme and thus decreases the level of platelet stimulant thromboxane A_2_ [5].

## 4. Why Aspirin Is Used as Prophylactic Agent against Thromboembolic Events

Aspirin was developed in 1897 and is one of the world's safest and cheaper drugs with proven efficiency for over 100 years [3]. A general practitioner in Glendale, California, named Lawrence Craven reported that daily low-dose aspirin has preventive role against myocardial infarction and stroke. He prescribed his 400 patients low-dose aspirin. None of his patients developed myocardial infarction for a period of 2 years (1948–1950) [6]. This was probably the first attempt to use aspirin to prevent myocardial infarction [3].

The prophylactic role of aspirin and other nonaspirin antiplatelet drugs has been confirmed by Anti-Platelet Trialists Collaboration after previous thromboembolic events. Vascular events were reduced by 20–25% in the first few years. The overall mortality rate was reduced by 12%. These results were based on a meta-analysis of 287 studies which involve a total of 135,000 patients [7]. Other studies and publications reported that antiplatelet treatment has reduced the overall mortality of vascular disease by 15% and nonfatal vascular complications by 30% [8].

## 5. Antiplatelet Dose of Aspirin

Aspirin is effective as antiplatelet drug at much lower doses than that required for analgesic and anti-inflammatory functions [9]. Antiplatelet activity of aspirin has been seen even at dose as low as 40 mg/day [10]. The antiplatelet properties are effective up to 320 mg daily dose [9]. In fact, doses of aspirin >320 mg/day may even decrease the effectiveness as antiplatelet agent due to inhibition of prostacyclin production [11].

Various evidence based studies recommended aspirin in the range of 75–100 mg/day for the prophylaxis against serious vascular events in high risk patients [9, 11]. However, recent randomized clinical trial indicates that 160 mg/day is the optimal dose of aspirin to prevent myocardial infarction and stroke [12]. In clinical situations where immediate antithrombotic effect is required (such as unstable angina, acute myocardial infarction, or stroke), a loading dose of 300 mg is recommended [11].

Most frequently recommended doses of aspirin for prevention of myocardial infarction and stroke are 81, 160, and 325 mg/day in the United States, whereas, in Europe and other countries, these doses are 75, 150, and 300 mg/day [12].

## 6. Risk of Continuing Aspirin Therapy prior to Surgery

When platelets activity is altered, a longer time period is required to stop bleeding from a cut surface because of alteration in primary hemostasis mediated by platelet plug formation [13].

Burger et al. reviewed 474 studies regarding the impact of low-dose aspirin on surgical blood loss. They stated that, in patients on aspirin, the average risk of intraoperative bleeding increases by a factor of 1.5 [14].

## 7. Risk of Stopping Aspirin Therapy prior to Surgery

It was recommended traditionally to stop the aspirin 7–10 days prior to invasive surgical procedure. However, there is scientific evidence which showed that stopping antiplatelet therapy is associated with a progressive recovery of platelet function and with a potential risk of rebound of thromboembolic vascular events. On stopping aspirin, there is excessive thromboxane A_2_ activity and decreased fibrinolytic activity [8].

Similarly, Ferrari et al. [15] and Chassot et al. [16] suspected the existence of a biological platelet rebound phenomenon on interruption of aspirin therapy, thus creating a prothrombotic state which may ultimately cause fatal thromboembolic events.

A systemic review and meta-analysis on the potential risks and health hazards of stopping aspirin confirmed the major detrimental impact and ominous prognostic implication of withdrawal across a greater number of individuals (50,279) at risk of de novo or recurrent cardiovascular events [17].

The adverse consequences of arterial thromboembolism are much more serious, as approximately 20% of these episodes are fatal and 40% episodes can lead to serious permanent disability [18]. In specific isolated circumstances, if stopping aspirin therapy is essential, it should be limited to 3 or fewer days. Risk of thromboembolic events increased considerably if aspirin therapy is discontinued between 4 and 30 days [15].

Retrospective studies showed potential risk of fatal thromboembolic events like myocardial infarction and stroke on stopping aspirin therapy [8, 15, 19]. The estimated risk of thromboembolic event if aspirin is stopped is approximately 1 in 21,448 cases [20].

Collet et al. performed retrospective analysis of 475 patients admitted to the hospital with the diagnosis of myocardial infarction. Eleven patients stopped aspirin therapy within 15 days prior to general surgical procedure. Another nine patients stopped aspirin 3 days prior to elective surgical procedures, one of which was a dental surgical procedure. This dental patient was stable and asymptomatic for a period of 10 years with continued use of aspirin. Unfortunately, myocardial infarction occurred 10 days after stopping aspirin therapy [21].

## 8. Debate Related to Timing for Stopping Aspirin Therapy

Aspirin alters the cyclooxygenase pathway and inhibits platelet activity. The effect of aspirin on platelets is irreversible. The effect of aspirin starts within 1 hour of ingestion and lasts for 7–10 days, that is, life span of a platelet [22, 23]. Therefore, traditionally it was recommended to stop aspirin therapy 7–10 days prior to invasive surgical procedure [24–28].

According to Daniel et al. [29] and Sonis et al. [30], antiplatelet therapy should be stopped 7 days preoperatively to minimize the risk of bleeding during surgery. Sonis et al. further stated that only the production of newer platelets will be able to overcome the inhibiting effect of aspirin. Therefore, stopping aspirin only for few days does not reverse the aspirin inhibition [30].

Sonksen et al. performed a clinical study on 52 healthy volunteers taking 7-day course of low-dose aspirin. They found that, after stopping the aspirin, the bleeding time (BT) was less than 10 minutes within 48 hours of stopping the aspirin therapy. Hence, they stated that withdrawal of aspirin for ≥5 days appears erroneous [31].

Wahl recommended that aspirin should be discontinued for 3 days only [32]. The rationale for such recommendation is that, after 3 days of interruption of aspirin, sufficient number of newer platelets (which are not affected by aspirin) will be present in the circulation for effective hemostasis [32, 33].

Ferrari et al. mentioned that, in specific isolated circumstances, if stopping aspirin therapy is essential, it should be limited to 3 or fewer days [15]. Medeiros et al. proposed that antiplatelet should be stopped 24 to 48 hours prior to surgery and restart 24–48 hours after surgery [34]. On the other hand, Conti recommended that there is no need to stop aspirin prior to invasive surgical procedure if bleeding time is within normal limit [35].

From review of literature, it can be concluded that there is considerable conflicting opinion regarding the exact time of stopping aspirin prior to invasive surgical procedure. Various authors recommended to stop aspirin preoperatively. The timing to stop aspirin ranges between 24 hours to 7–10 days. On the contrary some authors recommended not to stop aspirin preoperatively [15, 22–35].

## 9. Whether to Stop Aspirin Therapy prior to Invasive Surgical Procedure: Debate

The fear of excessive and uncontrolled intraoperative and postoperative bleeding prompts the medical practitioners to stop the aspirin before surgical procedures [1].

Few studies on patients on aspirin therapy undergoing cardiovascular surgery stated that there is risk of increased bleeding [36, 37]. Studies in the orthopedic, otolaryngology, and cardiology literature have shown increased bleeding in patients taking antiplatelet drugs [36, 38–40].

Few studies in the literature advocated stopping aspirin therapy prior to surgical procedure because of increased risk of bleeding [26, 28, 41]. However, if the aspirin therapy is altered or discontinued, there is increased risk of thromboembolic events [1, 28, 41]. Interestingly, none of these studies are from dental literature.

On the other hand, studies in dermatology literature concluded that there is no need to discontinue nonsteroidal anti-inflammatory drugs (NSAIDs) and aspirin preoperatively in patients undergoing cutaneous surgical procedure. There is no risk of increased bleeding in patients continuing their medications [42, 43].

Fijnheer et al. in a review article mentioned that there is scarcity of literature regarding cataract, dermatologic, ear, nose, and throat, and dental surgeries involving patients on aspirin medication [44].

Conti in an editorial mentioned that there is no need to stop aspirin prior to invasive surgical procedure if bleeding time is within normal limit [35]. Little et al. suggested that aspirin affected platelets did not cause significant bleeding complications unless the bleeding time is greater than 20 minutes [33]. Similarly, Sonksen et al. [31] and Gaspar et al. [45] claimed that there is no significant intraoperative and postoperative bleeding after dental extractions as long as prolongation in bleeding time remains within acceptable limit (bleeding time up to 20 minutes).

One has to maintain the balance between risk of increased bleeding and risk of thromboembolic events. On theoretical basis, stopping aspirin decreases the risk of bleeding but increases risk of thromboembolic events whereas continuing aspirin decreases risk of thromboembolic events but increases risk of bleeding. The ongoing debate is to find this balance based on the clinical evidences not solely on theoretical backgrounds. Also, carrying the information from different specialty surgical procedures with different degrees of invasiveness as compared to tooth extraction can be detrimental to patient's health. Therefore, review of dental literature is a must before coming to any definite conclusion.

## 10. Dental Literature regarding Increased Bleeding with Use of Aspirin Therapy

Schrodi et al. found increased bleeding on probing in patients who consumed aspirin in a dose of 325 mg/day for 7 days [46].

Royzman et al. studied the effect of 81 mg and 325 mg dose of aspirin on bleeding on probing. Fifty-four patients were divided into 3 groups. The first group consumed 81 mg aspirin daily for 7 days, second group consumed 325 mg aspirin daily for 7 days, and the third group consumed placebo for 7 days. They concluded that there is increased bleeding on probing in aspirin group, which can lead to impairment in diagnostic assessment and decision making for treatment planning [47].

Scher advocated stopping aspirin therapy before any elective surgery if it is not an emergency procedure. Continuous diffuse bleeding was present after surgery in patients taking aspirin therapy [27].

Similarly, a case was reported by Foulke in which bleeding episode occurred after oral prophylaxis with ultrasonic scaler [48].

Thomason et al. reported a case of excessive bleeding after a gingivectomy (in maxillary anterior segment) in a patient taking aspirin 150 mg/day. In this patient, platelet transfusion was required to control the excessive bleeding [49].

Elad et al. reported a case of severe bleeding episode following nonsurgical periodontal treatment in a patient taking dual antiplatelet therapy (aspirin 100 mg plus clopidogrel 75 mg/day). Preoperative values of platelet count and international normalized ratio (INR) were in normal range. Severe life threatening haemorrhage occurred postoperatively leading to haemorrhagic shock [50].

## 11. Dental Literature regarding Safety of Continued Aspirin Therapy prior to Tooth Extraction

Cañigral et al. conducted a study involving simple and surgical extractions in patients on antithrombotic therapy. The antithrombotic therapy comprises aspirin, clopidogrel, aspirin + clopidogrel, nonsteroidal anti-inflammatory drugs (NSAIDs), or low molecular weight heparin (LMWH). In 92% of instances, bleeding was stopped within 10 minutes with pressure alone. Only 8% of cases of moderate hemorrhage were easily managed by local hemostatic measures [2].

Brennan et al. reviewed the literature regarding the management of patients on aspirin requiring oral surgical procedures. They also recommended continuation of aspirin during dental extractions based on results of studies with high level of evidences [10].

Gaspar et al. concluded that ambulatory oral surgical procedures can be performed in patients on aspirin therapy as hemostasis posed no problem. Hence, they recommended continuation of aspirin therapy without interruption prior to oral surgical procedures [45].

Oral Medicine and Oral Surgery Francophone Society conducted a literature review and gave recommendations for management of patients on antiplatelet therapy based on the agreement among professionals in the field. The society stated that interruption of antiplatelet therapy prior to dental procedures is unnecessary. The risk of bleeding is very low and local hemostatic measures are usually successful [51].

A recent consensus opinion from the American Heart Association, American College of Cardiology, Society for Cardiovascular Angiography and Interventions, American College of Surgeons, and American Dental Association recommended either continuing aspirin and clopidogrel therapy for minor oral surgical procedures in patients who have coronary artery stents or delaying treatment until prescribed regimen will be completed [52].

Matocha stated that risk of bleeding after dental extractions is rare in patients on low-dose aspirin therapy. The incidence of postextraction bleeding complications, including other risk factors, does not exceed 0.2 to 2.3% [53].

Murphy et al. conducted a survey to assess the approach of dental practitioners in the management of patients taking warfarin and antiplatelet drugs. The results showed that, among the respondent dental practitioners, 86% of them who advised the patient to stop antiplatelet drugs prior to dental extractions do so with the consultation with the patient's physician. The authors stated that the protocol followed by dental practitioners in the management of patients on antiplatelet drugs and warfarin is not based on the current recommendations and guidelines [54].

Napeñas et al. conducted a retrospective analysis to evaluate the risk of bleeding complications in patients on single or dual antiplatelet therapy undergoing invasive oral surgical procedures including dental extractions. They concluded that risk of stopping antiplatelet therapy and predisposing the patient to thromboembolic events far overweighed the negligible risk of bleeding from dental procedures [55].

Ardekian et al. conducted a prospective study to evaluate the risk of bleeding after tooth extraction with the use of aspirin 100 mg/day. Suturing of the extraction socket and pressure pack had been used to achieve hemostasis in all the patients. Additional use of local application of tranexamic acid pressure pack was restricted in patients who presented with increased intraoperative bleeding. The authors found bleeding complication in patients who stopped aspirin as well as in patients on continued aspirin therapy. The bleeding incidence among two groups was comparable and the hemostasis was easily achieved with local hemostatic measures [56].

Nielsen et al. stated that minor dentoalveolar surgical procedures can be carried out safely without interrupting antithrombotic therapy if INR is within therapeutic range. Although aspirin and clopidogrel may increase the bleeding risk, the risk of fatal outcome is generally higher if treatment is stopped. They recommended use of local hemostatic measures and tranexamic acid mouthwash [57].

Allard et al. stated that the review of available literature is in favor of not stopping aspirin or clopidogrel in case of simple dental surgical procedures [58].

Hemelik et al. performed 151 tooth extractions in 65 patients on 100 mg/day aspirin therapy. The frequency of postoperative bleeding was 1.54% as compared to 1.59% in healthy control group. Higher frequency in control group is probably due to greater number of teeth extracted (543) in 252 patients. All bleeding episodes were handled easily. They concluded that there is no need to stop 100 mg/day aspirin prior to dental extractions [59].

Krishnan et al. in a prospective clinical study performed extraction procedures on patients taking aspirin, on patients who stopped aspirin preoperatively, and in normal patients [25 patients in each group]. Simple intra-alveolar extractions were performed and hemostasis was achieved with wet gauze pressure pack for 30 minutes. Patients were reassessed after 30 minutes for any persistent bleeding. After ensuring that hemostasis patients were discharged, patients were advised to report back in case of postoperative bleeding.

They categorized bleeding as clinically significant if it fulfills any of the following criteria:

1. bleeding that continued >12 hours of surgical procedure,
2. bleeding that makes the patient report back for management,
3. bleeding that results in large haematoma formation,
4. bleeding that required blood transfusion.

Krishnan et al. concluded that patient continuing aspirin therapy can undergo routine dental extractions without increased risk of excessive or prolonged bleeding [60].

The criteria of defining clinically significant bleeding after dental extractions in patients on antithrombotic therapy were suggested by Lockhart et al. [61] and were followed by various authors in subsequent studies.

Verma et al. performed a comparative study to evaluate the incidence of bleeding complications in patient on aspirin therapy. The study comprises three groups with 30 patients in each group. Group 1 patients on antiplatelet dose of aspirin (75–325 mg/day) continued it during extraction, Group 2 patients stopped their antiplatelet therapy, and Group 3 comprises normal patients not taking any antiplatelet drugs. Single tooth was extracted by intra-alveolar method in each group and the hemostasis was achieved in all cases by wet gauze pressure pack. The use of additional local hemostatic agents was restricted in case of bleeding complication. Criterion used to define bleeding as clinically significant was the same as suggested by Lockhart et al. [61] and used by Krishnan et al. [60]. None of the patients exhibited any excessive bleeding intraoperatively or immediately postoperatively. Delayed postoperative clinically significant bleeding was not present in any group. The authors concluded that single tooth extraction by intra-alveolar method in patient on continued aspirin therapy is a safe procedure [62].

Bajkin et al. conducted a prospective study to evaluate the postextraction bleeding in patients on aspirin monotherapy, oral anticoagulant therapy, and dual therapy with aspirin + oral anticoagulant (71 patients in each group). None of the patients on aspirin monotherapy has postoperative bleeding [63]. Although the primary aim of the study was to evaluate safety of dental extractions in patients on continued use of combined oral anticoagulant and aspirin therapy, the results related to aspirin therapy can be useful in the ongoing discussion.

Lillis et al. performed a prospective study to compare the incidence of bleeding complications among patients taking aspirin monotherapy, clopidogrel monotherapy, and dual therapy with both aspirin and clopidogrel and patients not taking aspirin at all. Out of 643 patients enrolled in the study, 111 patients were on antiplatelet therapy: aspirin monotherapy (*n* = 42), clopidogrel monotherapy (*n* = 36), and dual therapy with both aspirin and clopidogrel (*n* = 33). Patients not taking any antiplatelet drugs serve as control (*n* = 532). Teeth were extracted by simple method in all the patients under local anesthesia. To achieve hemostasis, extraction sockets were compressed with digital pressure for 2 minutes followed by sterile gauze pressure pack for 30 minutes. After 30 minutes, patients were reevaluated to look for any bleeding. If present, then a piece of oxidized cellulose (surgicel) was placed in the socket and suturing was done followed by sterile gauze pressure pack for 30 minutes and reevaluation. Patients were discharged after establishing hemostasis. Bleeding if present was categorized into the following headings.

*(1) Immediate Bleeding.* It is present at the time of extraction.

*(2) Prolonged Immediate Bleeding.* It persisted after 30 minutes of pressure pack and required use of surgicel, suturing, and second pressure pack with gauze.

*(3) Late Bleeding.* It is defined as clinically significant if it fulfills criteria mentioned by Lockhart et al. [61].

The results showed that greater number of patients on dual antiplatelet therapy showed prolonged immediate bleeding when compared to control healthy patient group and the difference is statistically significant. However, when incidence of prolonged immediate bleeding in patients on either aspirin monotherapy or clopidogrel monotherapy is compared with control group, the difference was not statistically significant. There is statistically significant difference when incidence of prolonged immediate bleeding was compared between dual antiplatelet therapy with aspirin monotherapy and clopidogrel monotherapy. No patient exhibited late bleeding. Although there is greater incidence of prolonged immediate bleeding in dual antiplatelet therapy group, hemostasis was achieved easily by local hemostatic measures. Therefore, they concluded that, the patient should not be predisposed to risk of thromboembolism by stopping either anti-platelet monotherapy or dual therapy [64].

In an editorial published in journal named Angiology, Koskinas et al. commented on the study conducted by Lillis et al. [64]. The authors mentioned that if the results of the study conducted by Lillis et al. were considered, it showed that the incidence of bleeding incidence was substantially (6-fold) higher in patients on antiplatelet monotherapy group compared with controls. If the dual therapy group was compared with control group, the bleeding incidence was increased by more than 100-fold. They further stated that if procedural safety was based merely on the incidence of bleeding complication, then the study of Lillis et al. might be interpreted as evidence of increased bleeding risk in patients on single and dual antiplatelet therapy particularly for dual therapy. However, if additional meaningful clinical parameters such as time frame of occurrence of increased bleeding and efficacy of local hemostatic measures to control bleeding are added, then the results can be interpreted differently; that is, patients on antiplatelet therapy have increased incidence of prolonged bleeding when compared to control group, but the increased bleeding was presented in the time frame of safe clinical setting and can be easily controlled by local hemostatic measures [65].

Shah et al. performed a prospective study to compare the incidence of bleeding complications among patients taking aspirin and those not taking aspirin at all. A total of 254 patients were enrolled in the study. Group 1 patients (*n* = 127) were taking 75–150 mg/day aspirin and continued it prior to extraction. Group 2 (*n* = 127) patients constitute the control group who were not taking any aspirin dose prior to extraction. One tooth was extracted by simple method in each patient of both groups. Hemostasis was achieved by similar protocol as described by Lillis et al. [64]. However, bleeding if present was categorized differently as follows.

*(1) Prolonged Immediate Bleeding.* It persisted after 30 minutes of pressure pack and required use of surgicel, suturing, and second pressure pack with gauze.

*(2) Late Bleeding.* It is defined as clinically significant if it fulfills criteria mentioned by Lockhart et al. [61].

*(3) Very Late Bleeding.* It is defined as continuous oozing at 24 and 48 hour follow up period.

The difference is that they have not considered intraoperative bleeding and added a new category as very late bleeding.

The results showed that 5 patients (3.93%) in aspirin group and 3 patients (2.36%) in control group presented with prolonged immediate bleeding which was managed by additional hemostatic measures. The difference was not statistically significant. Also 2 patients (1.57%) in aspirin group and 1 patient (0.78%) in control group presented with late bleeding at 12 hours postoperatively. This difference was again not statistically significant. Hemostasis was achieved easily by patients themselves with the help of pressure pack at home. None of the patients exhibited very late bleeding. They concluded that it is a safe practice to perform simple extraction of 1 tooth in patients taking 75–150 mg aspirin daily [66].

Madan et al. performed minor oral surgical procedures in patients on low-dose aspirin therapy (75–100 mg/day). The surgical procedures performed were simple and surgical extractions and implant placement. Suturing and pressure pack for 30 minutes had been used as hemostatic measure in all the cases. After 30 minutes, patients were reassessed for hemostasis. Intraoperative blood loss >30 mL was considered excessive. The results showed that only 1 patient after 3rd molar extraction showed excessive bleeding intraoperatively which was easily managed by pressure pack soaked in 1% feracrylum solution. There was no postoperative bleeding in any case. These authors concluded that most minor oral surgical procedures can be carried out safely without interrupting long-term low-dose aspirin therapy [67].

Aframian et al. conducted a retrospective analysis of studies published on management of dental patients taking common hemostasis altering medications. After critical analysis of literature, they recommended that low-dose aspirin therapy (≤100 mg/day) should not be stopped prior to dental procedures. Local hemostatic measures are generally effective to control intraoperative and postoperative bleeding if present. They further stated that the benefit of continued aspirin therapy to prevent thromboembolic event clearly outweighs the risk of increased bleeding episode. This is a Class-I recommendation based on Level of Evidence B [68].

Park et al. performed a prospective clinical study to evaluate the safety of dental extractions in patients on continued antiplatelet therapy with multiple drugs. Fifty-nine patients were on dual aspirin (100 or 200 mg/day) + clopidogrel 75 mg/day therapy and 41 patients were on triple antiplatelet therapy (aspirin 100 or 200 mg/day plus clopidogrel 75 mg/day plus cilostazol 100 mg/day). A hundred patients not taking any antiplatelet agents served as control group. Only 3 patients exhibited post-operative bleeding (1 on dual, 1 on triple anti-platelet therapy and 1 not taking any anti-platelet drug). All the episodes of bleeding were easily controlled by pressure application by patients themselves. The authors concluded that dental extractions can be performed safely in patients on multiple antiplatelet agents [69].

A prospective trial conducted by Cardona-Tortajada et al. involving 155 patients on antiplatelet therapy confirmed that local measures to achieve hemostasis are sufficient to control postoperative hemorrhage after tooth extraction. It is advisable to minimize the surgical trauma by minimizing the number of teeth to be extracted at a time. It has been recommended that three single rooted teeth and two molars either adjacent or corelative to each other should be extracted during a single visit [70].

Morimoto et al. conducted a prospective clinical study involving patients on antithrombotic therapy. Three groups involved in the study were patients on warfarin monotherapy (*n* = 134), patients taking antiplatelet monotherapy (87), and patients taking combination therapy with warfarin and aspirin (*n* = 49). Intra-alveolar extractions of teeth were performed followed by placement of oxidized cellulose in extraction sockets and suturing in all cases to achieve hemostasis. A total of 513 teeth were extracted on 306 occasions. The reported incidence of postoperative bleeding was 3.6%. It includes 7 patients on warfarin monotherapy and 2 on combination therapy. No patient on aspirin monotherapy showed postoperative bleeding. The authors concluded that hemostasis can be achieved easily after tooth extraction in patients on warfarin (INR < 3.0) and antiplatelet therapy [71].

Partridge et al. performed prospective observational study to evaluate the effect of platelet altering medications on bleeding from minor oral surgical procedures. The two groups involved in the study were experimental group taking platelet altering medications and control group in which platelet altering medications were stopped at least 10 days prior to surgery. The minor oral surgical procedures performed were simple and surgical extractions of the teeth, alveoloplasty, biopsy, and frenectomy. The results showed that there is no statistically significant difference in relation to amount of blood loss per unit surgical area in both groups. The authors recommended not to alter antiplatelet medications prior to minor oral surgical procedures [72].

Nooh performed simple and surgical extractions in patients on aspirin therapy (81 mg/day). Normal patients that had undergone the same procedures served as control group. Wet gauze pressure pack for 30 minutes and figure of 8 suturing were adequate to achieve hemostasis in simple and surgical extractions, respectively. Patients on aspirin therapy undergoing surgical procedure exhibited mild oozing easily controlled by pressure packs alone. Therefore, the author concluded that patients taking 81 mg of aspirin can undergo dental extractions and there is no clinically significant increased bleeding risk [73].

Duygu et al. performed a clinical study to assess the effect of antiplatelet drugs on risk of bleeding complications after teeth extractions. Simple dental extractions were performed in experimental group patients on continued aspirin therapy (*n* = 25) and control group patients who stopped aspirin 7 days prior to extractions (*n* = 19). The experimental group patients were on aspirin dose in the range of 75–300 mg. Method to achieve hemostasis ranges from wet gauze pressure pack, locally applied tranexamic acid, to the use of gelatin sponge and suturing. Local hemostatic measures were able to maintain primary hemostasis in all cases. There were no intraoperative and postoperative bleeding complications in any case including the patients with prolonged BT of 12.3 minutes. There was no statistically significant difference between two groups with respect to postoperative bleeding complications. They concluded that there is no need for interruption of long-term aspirin therapy prior to dental extractions [74].

In a review article, Kumar et al. stated that current consensus and recommendations favor not to stop antiplatelet therapy prior to invasive dental procedures. They further advised to take precautions in these patients during treatment because other confounding risk factors may complicate the problem [3].

In a review article, Badal et al. stated that current researches suggested that there is no indication to stop aspirin therapy prior to invasive dental procedures as any postoperative bleeding if present can be easily managed by local hemostatic measures [75].

Medeiros et al. performed simple single tooth extraction (molar tooth) in patient on aspirin therapy. Sixty-three patients involved in the study were randomly divided into two groups. Group S patients (*n* = 31) have suspended their aspirin therapy 7 days prior to extraction. Group NS patients (*n* = 32) have not suspended their aspirin therapy (100 mg/day) and undergone extraction. Suturing was used as a hemostatic measure in all the patients. Additional use of biological adhesive was restricted to those patients who showed increased intraoperative bleeding as compared to others. None of the patients exhibited any bleeding complication postoperatively. The results showed that mean (±standard deviation) volume of blood loss per tooth extraction was 16.38 ± 13.54 mL in group NS (aspirin not suspended) and 12.10 ± 9.37 mL group S (aspirin suspended). Although the mean blood loss per tooth extraction was greater in group NS, this was not statistically significant. Based on these results, they concluded that there is no need to suspend aspirin therapy (100 mg/day) prior to single molar extraction [76].

Brennan et al. performed a double blind randomized controlled study in healthy patients. A total of 36 healthy patients were randomly divided into two groups. Group 1 patients (*n* = 17) were given aspirin 325 mg/day two days preoperatively and continued for further 2 days after extraction (total 4 days). Group 2 patients (*n* = 19) received placebo with similar time schedule as followed in Group 1. Single tooth extractions were performed in both groups. The two groups were similar with respect to baseline information, site of extraction, extraction difficulty, extraction time, and compliance. The primary outcome was intraoral bleeding time and the secondary outcome was bleeding complication. Although intraoral bleeding time was greater in aspirin group, it is not statistically significant. Also, there was no statistically significant difference between two groups with respect to postoperative bleeding. They concluded that there is no indication to stop aspirin in patients requiring single tooth extraction [77].

Garnier et al., in a retrospective analysis of 52 patients, reported that tooth extraction can be performed without stopping aspirin therapy. A total of 218 teeth were extracted without stopping aspirin therapy. Suturing and pressure pack were used as hemostasis measure in all cases. Only one patient (1.9%) (three extraction sockets (1.3%)) presented with continuous bleeding which required additional local hemostatic measure. No systemic hemostatic measure was required. They concluded that the haemorrhagic risk in patients on aspirin therapy can be managed by local hemostasis protocol [78].

## 12. Discussion

Decision to continue or stop the antiplatelet therapy is like weighing risk of thromboembolic event against risk of bleeding. Before decision making, some factors need consideration. These factors are patient's inherent risk factors for bleeding, additional ongoing treatment which increases the bleeding risk, invasive potential of the surgical procedure, and potential risk of thromboembolic event if antiplatelet therapy is stopped [79]. In addition to these, previous history of bleeding episode, haemorrhagic peptic ulcers, or haemorrhagic stroke increases possibility of bleeding [8].

Patient's inherent factors which can increase the risk of bleeding must be identified prior to invasive surgical procedure. Patient's demographic risk factors include advanced age and female sex. Additional patient related risk factors include obesity, hypertension, diabetes mellitus, haemostatic disorders, renal impairment or failure, and other major organ system failures [8, 80].

Only considering the positive studies in favor of continuing aspirin and ignoring the potential risk associated with aspirin use is not a sound scientific principle. Therefore, a discussion regarding negative reports indicating potential risk of bleeding with aspirin use is essential.

Schrodi et al. [46] and Royzman et al. [47] reported increased bleeding on probing in patients on aspirin therapy. Similarly, Lillis et al. [64] and Shah et al. [66] reported that bleeding complications after tooth extraction (in patients on aspirin) were slightly higher if tooth extraction was performed because of periodontitis as compared to dental caries.

Lillis et al. further stated that local inflammation (in relation to site of tooth extraction) is characterized by hyperemia along with possible fragility of blood vessels that might predispose to postextraction bleeding. Therefore, patients taking aspirin therapy and requiring extraction of periodontally involved teeth must be considered as high risk group, with more probability to develop bleeding postoperatively. Appropriate hemostatic measures should be used in these patients [64].

From the above description, it can be concluded that increased bleeding on probing as reported by Schrodi et al. [46] and Royzman et al. [47] was not solely due to aspirin use. Local risk factor (periodontitis) leads to hypermeia which adds to increased incidence of bleeding on probing in these patients.

Elad et al. reported a case of severe life threatening bleeding episode following nonsurgical periodontal treatment in a patient taking dual antiplatelet therapy. This 56-year-old female patient has multiple systemic illnesses like ischemic heart disease, hypertension, and diabetes [50]. Advanced age, female sex, hypertension, and diabetes are patient's inherent risk factors [8, 80] and periodontal problem is local risk factor [64] which were not at all considered by the authors. The authors should have considered additional local and systemic measures preoperatively to avoid such life threatening complication. With respect to severity of the complication, we may consider it as a single case of significant postoperative complication.

Foulke et al. reported a case of postoperative bleeding episode which occurs after oral prophylaxis with ultrasonic scaler. They linked this bleeding complication with the use of aspirin [48]. However, the patient has taken double dose of aspirin the night before surgery [76]. Also, oral prophylaxis is required for removal of plaque and calculus responsible for gingival and periodontal disease, which is a local risk factor for increased bleeding tendency (as already explained) [64].

Scher found continuous diffuse bleeding after surgery in patients taking aspirin therapy [27]. However, the patients involved in their study were taking much higher doses of aspirin [3, 76].

Case reported by Thomason has excessive postoperative bleeding after gingivectomy in maxillary anterior segment. This patient was treated by renal transplant and had decreased platelet count [49]. Renal failure is considered as patient's inherent risk factor which predispose to postsurgical bleeding (not considered by the author) [8, 80]. Therefore, it is difficult to consider altered platelet activity induced by aspirin as the single responsible factor when other risk factors like decreased platelet count and renal failure were also present. In the same patient, initial gingivectomy procedure in mandibular anterior region was uneventful. It is not clear how the same patient with same invasiveness of surgical procedure responded differently.

Moreover, the dental literature regarding risk of bleeding associated with use of aspirin was based on isolated case reports or small case series, whereas literature regarding the safety of tooth extraction in patients on continued aspirin was based on studies with adequate sample size.

## 13. Conclusion

Extraction is one of the most common procedures performed in oral surgery. Surgical procedures performed on the patients must be based on sound scientific knowledge of literature. Nothing is static, so is the science. Recommendation changes from time to time. Based on the review of literature, it can be concluded that current recommendations and consensus are in favor of not stopping antiplatelet dose of aspirin prior to tooth extraction. The safety of dental extractions in such patients is supported by studies reported in literature. It must be emphasized that appropriate use of local hemostatic measures should always be considered whenever indicated. There is no justification to predispose the patient to the risk of thromboembolism at the expense of minor bleeding which can be easily controlled.
